# Late-career workforce participation in times of rising state pension age: the role of health and motivation

**DOI:** 10.1186/s12889-025-25556-1

**Published:** 2025-11-21

**Authors:** Dorly J. H. Deeg, Maaike van der Noordt, Astrid de Wind, Cécile R. L. Boot

**Affiliations:** 1 Department of Epidemiology and Data Science, Amsterdam UMC, Vrije Universiteit Amsterdam, Amsterdam Public Health Research Institute – Ageing & Later Life Programme, P.O. Box 7057, 1007 MB Amsterdam, The Netherlands; 2 Department of Public Health Foresight, Center for Public Health, Healthcare and Society, National Institute for Public Health and the Environment (RIVM), P.O. Box 1, 3720 BA Bilthoven, The Netherlands; 3 Department of Public and Occupational Health, Amsterdam UMC, University of Amsterdam, Amsterdam Public Health Research Institute – Societal Participation & Health programme, P.O. Box 7057, 1007 MB Amsterdam, The Netherlands; 4 Department of Public and Occupational Health, Amsterdam UMC, Vrije Universiteit Amsterdam, Amsterdam Public Health Research Institute – Societal Participation & Health Programme, P.O. Box 7057, 1007 MB Amsterdam, The Netherlands

**Keywords:** Late-career workers, State pension age, Health, Motivation, Time trend

## Abstract

**Background:**

This study addresses late-career workforce participation and its association with health and motivation during nine years in which state pension age (SPA) rose gradually from 65 to 66.6 years in the Netherlands.

**Methods:**

Using the Longitudinal Aging Study Amsterdam, we studied workers aged 61–63 years who at 3-year follow-up had reached ages 64 years up to SPA, in 2013–2016 (*n* = 82), 2016–2019 (*n* = 111), and 2019–2022 (*n* = 119). Workforce participation was defined as continued working and number of working hours/week. Physical and mental health included self-rated health, functional limitations, depressive symptoms, and cognitive ability. Motivation consisted of self-reported reasons for (change in) workforce participation. Logistic (continued working) and linear (working hours) regression models were controlled for age, sex, educational level, and partner status.

**Results:**

Over time, 58% (2013–2016), 82% (2016–2019), and 72% (2019–2022) continued working. Among the health indicators, only better self-rated health predicted continued working, and only in 2019–2022. In continuing workers, working hours remained stable around 31 h in 2013–2016 and 2016–2019, but decreased to 26 h in 2019–2022. Poorer physical health predicted a decrease in working hours only in 2013–2016. Only in 2019–2022, better mental health was significantly associated with a reduction in working hours. Exited workers and workers who had reduced their working hours reported a lack of motivation to work more often in 2022 than in 2019.

**Conclusions:**

In a period of rising SPA, an increasing share of workers aged 61-SPA continued work participation. Health played a minor role, whereas motivation to work became more important. Our findings suggest that the feasibility of maintaining late-career workers in the workforce requires attention to both health maintenance and enhancement of motivational factors at work.

**Supplementary Information:**

The online version contains supplementary material available at 10.1186/s12889-025-25556-1.

## Introduction

Like many governments of industrialized countries, the Dutch government is raising the state pension age (SPA), i.e. the age at which Dutch citizens are entitled to receive state pension. The SPA is legally linked to the statutory retirement age, i.e., by default the age at which labour contracts are ended [1]. Whereas in the Netherlands a SPA of 65 years was in place since the late 1950 s up to 2012, between 2013 and 2024 the SPA was raised to the age of 67 years and is planned to continue to rise beyond the age of 67 years, indexed to increases in life expectancy. As in other countries, this measure provoked protests, organized by trade unions and senior citizens' associations, stemming from concerns about the feasibility of everyone having to work longer, including those experiencing health problems or poor working conditions. The protests also called workers’ willingness to extend working life into question [2]. The current study addresses the feasibility of extending working life by examining to what extent health and the motivation to work affect workforce participation of late-career workers.

Already in the decade prior to the raise of SPA, the Netherlands ranked among the countries with the steepest rise in workforce participation among late-career workers [3, 4]. In this period, the Dutch government introduced a series of policy reforms, including abolishment of early retirement regulations and restriction of access to work disability and unemployment benefits. These regulations reached their full effects on workforce participation in the early 2010 s, when also the SPA was starting to rise [5].

The effect of raising the SPA has been addressed by examining whether late-career workers are actually remaining in the workforce up to the SPA or alternatively, if they exit from the workforce earlier by other routes. Findings are not unambiguously positive. A French study using registration data found that, despite a sizable positive effect on the employment rate, the reform also led to increased unemployment and work disability rates [6]. Likewise, a Dutch study using registration data between 2013 and 2018, showed an increase in the number of workers leaving the labour market through unemployment or work disability [7]. These studies, however, did not link their data with the health and motivation of individual workers.

Longitudinal studies provide evidence that poor health is a major limiting factor for workforce participation, with particularly strong association with work disability and unemployment [8–10]. The majority of studies have focused on current workers to prospectively predict continuation in versus exit from the workforce. However, workforce exit is only one aspect of workforce participation. Considering that workforce participation is the amount of time spent on paid work, and thus contributing to the national product, hours worked should be examined as well [11]. If workers stay in the workforce but work shorter hours, it partly undermines the policy aim of extending working lives. Studies addressing the role of health in the number of working hours show a positive association between good health and hours worked [11–13]. Among the studies on the prospective association of health with workforce participation, to the best of our knowledge, no study addressed changes over time in this association. This, despite the fact that several studies had follow-up periods of more than a few years and that new policy measures to extend working lives may have been implemented during these periods.

In the studies performed so far, various health indicators were used. While most studies use self-rated health as a single question, several studies additionally use chronic morbidity, physical limitations, and/or mental health [9, 10, 14, 15]. Objective health indicators are rarely used [11]. Although cognitive ability is of increasing relevance for workforce participation [16], only very few studies included cognitive health, showing associations with subjective work ability and exit through work disability [17–19]. In accordance with recent recommendations [20], we defined health broadly including four subjective and objective indicators: self-rated health, functional limitations, depressive symptoms, and tested cognitive ability.

In addition to health, motivation to work plays a key role in work participation [21–23]. Motivation to work has been conceptualised as playing a role in the association between health and workforce participation [20]. In particular, poor health may not lead to workforce exit in the presence of a strong, intrinsic motivation to work. Intrinsic motivational factors include attachment to the work organisation, perceived meaningfulness of the tasks, the desire to help others, and to keep using one’s skills [22–27]. In addition, financial concerns as an extrinsic motivational factor may postpone retirement [26]. These motivational factors may support continued employment even when physical or mental health is impaired [20, 28, 29]. Vice versa, when the motivation to work is low or when factors outside work are considered more important, workforce exit may occur even in the presence of good health. For example, due to changes in life circumstances, the work-life balance may change. Many workers consider retiring before the SPA in order to have more leisure time [24, 25]. In this study, we examine how workers nearing SPA motivate changes in their workforce participation and to what extent their motivations are linked to their health.

Earlier studies include relatively broad age ranges, mostly workers aged 50 years and over. However, work motivation in workers who are a few years from SPA, as well as the association between health and motivation to work, may differ from workers for whom SPA is further in the future [27].

The actual average retirement age of Dutch workers increased during our study period from 64 years in 2013 to 66 years in 2022, which is still about a year earlier than the SPA [5]. Since the 1980’s, the workforce participation of workers aged 64 up to SPA has not been as high and is likely to continue to grow in the near future. Therefore, it is highly relevant for pension policy evaluation to investigate how workforce participation in this age group has developed in the successive years in which the statutory retirement age has been rising, in terms of both continuation of working life and hours worked, and to which extent health and motivation have been of influence. Thus, this study focuses on workforce participation in age groups 61–63 years that had reached age 64 but were still younger than SPA at three-year follow-up, in three successive periods (2013–2016, 2016–2019, and 2019–2022).

Research questions:

1. To what extent does workforce participation (continuing paid work, number of hours worked) change between 2013 and 2022 in the age group 61-SPA?
2. To what extent do health indicators predict three-year change in workforce participation, and does their predictive ability change over time between 2013 and 2022?
3. How do late-career persons motivate their (change in) workforce participation, and to what extent are these motivations associated with health?

We expect that the rise in SPA leads late-career workers to stay in the workforce up to older ages. As poor health is increasingly common with advancing age and thus with extended working lives, we initially expect that in successive cohorts of late-career workers health plays an increasing role regarding the continuation of workforce participation and the number of hours worked. However, there is recent evidence that continuing working up to older ages is accompanied by an increase in working years spent in poor physical health among workers [30–32]. This increase is a likely consequence of the lesser availability of financially feasible exit routes from the labour market, even for workers in poor health. Additionally, an increase in working years may be facilitated by the general rise in educational level and by technological and organizational changes that involve a decrease of physical labour and an increase in labour that requires cognitive rather than physical effort [16, 33, 34]. Due to informatisation, however, cognitive demands have increased, which may compromise mental health [35, 36]. Consequently, the role of health in workforce participation over time is not clear-cut.

As the third period of our study for a large part coincides with the COVID-19 pandemic, an additional dynamic was introduced. Recent studies show that COVID-19 containment measures, such as changes in the organisation of work, but also reconsideration of the work-life balance, may lead to working fewer hours or to earlier retirement [37–40]. We expect that motivation to work plays a larger role in this period than in earlier periods.

## Methods

### Sample and design

Data were used from the Longitudinal Aging Study Amsterdam (LASA), an ongoing, prospective cohort study based on representative samples of the older population in the Netherlands [41]. The first cohort was recruited in 1992/93, including 3107 participants aged 55 to 85 years. After 10 and 20 years, refresher cohorts aged 55 to 64 years were recruited, including 1002 participants in 2002/03 and 1023 participants in 2012/13. In each cohort the baseline cooperation rate was similar at 62%. Measurement waves are conducted every three years and include a face-to-face computer assisted interview in the home of the participant or a short telephone interview for participants who decline a face-to-face interview.

The current study focused on participants in the third cohort, which started in 2012/13. Comparisons were made across three periods^1^: 2013–2016 (period 1), 2016–2019 (period 2), and 2019–2022 (period 3). Participants were included who did paid work at the beginning of each period and had not yet reached SPA after 3 years. As the minimum age was 64 years at the end of period 3, for comparability across periods a further selection criterion was that participants’ minimum age was 64 years at the end of each period. These criteria yielded separate samples of 82, 111, and 109 eligible participant at baseline of the successive periods. Because of study attrition, in each successive period 64, 99, and 97 participants remained, respectively. Attrition was significantly greater in period 1 than later on, was marginally significantly greater in workers in less-than-good self-rated health, and not significantly associated with any other study variable (Supplement Table 1).

### Measures

#### Workforce participation

At both baseline and follow-up, work state was coded as 1: having paid work, i.e., working one or more hours per week, and 0: not having paid work. Working hours were self-reported as number of hours actually worked weekly.

#### Health indicators

Functional limitations were based on questions about difficulty in performing six activities, including walking up and down stairs; using own or public transportation; cutting own toenails; dressing and undressing; sitting down in and standing up from a chair; and walking outside for five minutes without stopping [42]. The response options were 0: yes, without difficulty; 1: yes, with some difficulty; 2: yes, with much difficulty; 3: only with help; 4: no, I cannot. The summed score ranged from 0 to 24, with higher scores reflecting more limitations.

Self-rated health was measured by asking the widely-used question: “How is your health in general”, with scores from 1: very good to 5: poor.

Depressive symptoms were ascertained using the Dutch translation of the 20-item Center for Epidemiologic Studies Depression scale [43, 44]. Participants were asked to indicate how often during the past week they had experienced each symptom with response categories 0: (almost) never to 3: (almost) always. The summed score range was 0–60, with higher scores reflecting more depressive symptoms.

Cognitive ability was measured using an abbreviated version of the Mini-Mental State Examination adapted for a telephone interview, testing orientation in time and place, attention, and working memory [45, 46]. Scores ranged from 0–16, with higher scores indicating better cognitive ability.

All health indicators showed non-linear associations with the outcome measures, and were therefore dichotomised at the inflection point. Thus, cut points were set between 1 and 2 for functional limitations; between 2 and 3 for self-rated health; between 15 and 16 for depressive symptoms; and between 14 and 15 for cognitive ability. For each health indicator, good health was coded as 0 and poor health, as 1.

#### Motivation for (change in) work participation

Starting from 2019, different sets of motives were presented to participants depending on whether they had exited from or continued in the workforce in the past three years [47]. In 2019, participants could select one among a list of motives; in 2022, they could indicate more than one motive. Lists of possible motives were provided, with an additional category ‘other’ leaving participants space to fill in their own reason. The ‘other’ responses were either categorised with already listed reasons, or kept as a newly labelled category.

Workers who reported to have retired (as opposed to exit due to unemployment, work disability, or inactivity) were asked for reasons for exit. These were combined into four categories: D: work demands and/or health (work pressure too high, work physically too demanding, health problems too limiting), M-: lack of motivation to work (organizational changes or conflicts at work, no longer motivated, lost interest, financially no longer necessary, worked long enough), F: financially more favourable, and P: work-life balance (more enjoyable to spend more time on private life, providing informal care, partner is retired, more time needed for activities outside work, enjoy life as long as health permits, more freedom, less obligations).

Among continuing workers it was asked whether a participant who was in the workforce would partially or completely retire if this was made financially possible. Reasons to (partially) retire included the same categories as reasons for exit. Reasons not to retire were combined into three categories: M +: motivation to work (enough challenges at work, social contacts at work), F: financial benefit, and L: loyalty to others (request of employer or colleague, partner is still working).

#### Covariates

In order to control for potential compositional shifts in the workforce across the study period, we included several socio-demographic covariates. Age and sex were derived from the population registry. Educational level was self-reported as the highest level of education completed, categorised as 1: low (up to lower vocational education), 2: intermediate (intermediate vocational to general secondary education) or 3: high (tertiary and higher education). Partner status was self-reported as 0: no partner; 1: partner, cohabiting; and 2: partner, not cohabiting.

### Statistical analyses

First, study variables were compared between the three periods and between work states. Period was included as a categorical variable. To test differences between periods and work states, analysis of variance was used for continuous variables and logistic regression, for categorical variables.

To address the predictive value of health for work continuation, a series of logistic regression analyses was performed in participants working at baseline, with dependent variable work state at follow up. In order to maximise power, each model included one health indicator. Interaction terms of health and period were calculated to determine period differences in the association of health and work state.

For the study of the predictive value of health for change in number of hours worked, Generalised Estimating Equations (GEE) were used with period and within-person time as independent variables. Within-person time was coded for each period as 0: baseline and 1: follow-up. A series of linear regression models included one health indicator each as the main independent variable. Each model consisted furthermore of three two-way interaction terms, i.e., within-person time*period, health*period, and health*within-person time, and the three-way interaction term of health*within-person time*period. By changing the reference category of the period variable, we estimated a model for the health*within-person time interaction for each period, with the covariates having equal effects [48].

Covariates in all logistic and GEE models were age, sex, educational level, and partner status. In preliminary models of work continuation, working hours was included as a covariate, but proved far from significant (*p* > 0.4) and was dropped for reasons of parsimony.

Main effects were considered significant at *p* < 0.05; interaction terms were considered significant at *p* < 0.10 [49].

Analyses regarding motivations were restricted to frequency counts and bivariate associations with work participation and health, because of the small sizes of the subsamples in which different sets of motives were asked.

## Results

### Baseline characteristics

Table 1 shows baseline socio-demographic and health characteristics by work state for each of the three periods. In the successive samples, the participants’ SPA rose from average 65.7 to 66.8 years. Correspondingly, their average baseline calendar age rose slightly from 61.6 to 62.5 years. Calendar age did not differ between workers and non-workers. Workforce participation amounted to 59%, 58% and 73% at baseline of the successive periods, which rise proved non-significant. Women and lower educated respondents were overrepresented among non-workers across the periods. No significant differences were found regarding partner status.

**Table 1:** Participants aged 61–63 years at baseline and 64 years up to SPA at follow-up: baseline characteristics of paid workers and non-paid workers in 2013, 2016, and 2019 (column percentages)

Statutory retirement age (M,sd)	Period 1, baseline 2013		Period 2, baseline 2016		Period 3, baseline 2019		
	65.7 (0.2)		66.3 (0.0)		66.8 (0.2)		
	Paid work: 59% (*n* = 64)	No paid work: 41% (*n* = 45)	Paid work: 58% (*n* = 99)	No paid work: 42% (*n* = 73)	Paid work: 73% (*n* = 97)	No paid work: 27% (*n* = 35)	Signifcance^†^
*Individual characteristics at baseline*							
Age (M,sd)	61.6 (0.5)	61.6 (0.5)	62.0 (0.7)	62.2 (0.7)	62.5 (0.6)	62.4 (0.6)	a
Gender (%):							b
Male	58	38	59	47	50	37	
Female	42	62	41	53	50	63	
Education (%):							b
Lower	20	38	25	33	19	31	
Intermediate	47	38	35	45	43	46	
Higher	33	24	39	22	38	23	
Partner (%):							-
Cohabiting	80	67	82	82	80	63	
Not cohabiting	3	4	4	1	5	11	
None	17	29	14	16	14	26	
Functional limitations (%)	9	38	14	31	9	46	b
Less-than-good self-rated health (%)	30	51	16	23	27	57	a,b
Depressive symptoms (%)	8	27	8	11	3	23	b
Cognitive impairment (%)	23	28	18	19	8	24	a
*Work participation*							
Paid work at 3-year follow-up (%)	58	2	82	15	72	-	a,b
Hours work/week at baseline (M,sd)	30 (11)	-	32 (14)	-	30 (13)	-	
1–19 h (%)	16		18		20		
20–31 h (%)	31		20		29		
32–39 h (%)	30		26		24		
40–70 h (%)	23		35		27		
Hours work/week 3-year at follow-up (M,sd)	31 (13)	6 (-)^a^	32 (14)	8 (5)	26 (13)	-	a
1–19 h (%)	19		17		28		
20–31 h (%)	33		25		37		
32–39 h (%)	17		21		16		
40–70 h (%)	31		37		19		

^†^a: period difference is significant (*p* < 0.05); b: work state difference is significant (*p* < 0.05)

^*^The periods are indicated by single year-at-baseline; indicated is the second year of a wave, as in all waves, the majority of interviews was held in the second year

^a^No SD, because *n* = 1

Among the health indicators, functional limitations and self-rated health were significantly better in workers than in non-workers. Also, workers had significantly fewer depressive symptoms, but not better cognitive ability than non-workers. Self-rated health was significantly better in period 2 than in periods 1 and 3. Cognitive impairment decreased across the periods, particularly among workers. The correlations between the four health indicators were modest (*r* = 0.03–0.50) (Supplement Table 2).

Among workers at baseline, the percentage continuing working at 3-year follow-up increased significantly from 58% in 2016 to 82% in 2019 and decreased somewhat to 72% in 2022. The number of weekly working hours at baseline was stable across the periods at around 31. At follow-up the continued workers’ hours worked remained about the same in periods 1 and 2, but declined significantly to 26 h in period 3. Particularly workers who worked full-time or longer in 2019 had reduced their hours substantially in 2022.

### Baseline health predictors of work status at follow-up

#### Continued working

Pooling the three samples, the variable ‘period’ proved significant, i.e., in periods 2 and 3 continued working was more likely than in period 1.

None of the baseline health indicators predicted continued working versus exit from the workforce as a main effect (Table 2). However, self-rated health showed a significant interaction with period 3 versus periods 1 and 2. Whereas in periods 1 and 2 the odds ratios (ORs) of continuing working were far from significant at 0.73 (95% Confidence Interval [95%CI] 0.23–2.36) and 1.64 (95%CI 0.32–8.32), in period 3 the OR was 0.20 (95%CI 0.07–0.54), indicating that less-than-good self-rated health prevented participants from continuing working in this period.

**Table 2:** Associations (Odds Ratios, 95% Confidence Intervals) of four dichotomous health indicators with continuing versus stopping paid work within 3 years in workers aged 61–63 years at baseline. Logistic regression adjusted for age, sex, education, partner status, and period; each row is one model. In case of a significant interaction effect of health indicator*period, the ORs are presented for each period

	OR	95% CI
Functional limitations^a^	0.69	0.27–1.78
Self-rated health^b,†^:		
—Period 1	0.73	0.23–2.36
—Period 2	1.64	0.32–8.32
—Period 3	0.20	0.07–0.54*
Depressive symptoms^c^	1.07	0.30–3.76
Cognitive impairment^d^	1.13	0.49–2.64

^†^Interaction self-rated health*period significant at p = 0.06

^a^Score >= 2

^b^Less than good

^c^Score Center for Epidemiologic Studies Depression scale >= 16

^d^Score 9-item MiniMental State Examination < = 14

*p < 0.05

A majority of respondents who were retired from the workforce reported as a reason the wish to have more time for private life, followed by issues with work demands or health. A lack of motivation to work was reported as a reason in 2022 only (Table 3, first panel). The reason ‘work demands or health’ was associated with functional limitations, a lack of motivation was associated with less-than-good self-rated health, and the wish for more time for private life was inversely associated with functional limitations. No other motivation-health associations proved significant (Supplement Table 3).

**Table 3:** Motivations for (changes in) workforce participation in 2019 and 2022: absolute numbers and, to facilitate comparison, percentages within one year between brackets

AGGREGATED CATEGORIES OF REASONS	2019	2022^a^
*Exited workers when (partly) retired*		
*N*	*8*	*14*
Demands/health (D)	2 (25%)	7 (27%)
Lack of motivation (M-)	0	9 (35%)*
Finances (F)	0	0
Private life (P)	6 (75%)	10 (38%)
*Continuing workers who would want early retirement if made financially possible*		
*N*	*40*	*23*
Demands/health (D)	17 (42%)	10 (32%)
Lack of motivation (M-)	5 (13%)	3 (10%)
Finances (F)	1 (3%)	2 (6%)
Private life (P)	17 (43)	16 (52%)*
*Continuing workers who would want to continue working if retirement was made financially possible*		
*N*	*35*	*31*
Motivation to work (M +)	26 (74%)	26 (72%)
Finances (F)	4 (11%)	5 (14%)
Loyalty to others (L)	2 (6%)	5 (14%)

D includes: Work pressurre too high, Work physically too demanding, Health problems too limiting

M- includes: Work-related factors (organizational changes, conflicts); No longermotivated/lost interest; Financially no longer necessary; Worked long enough

F includes: Financially more favourable

P includes: More enjoyable to spend more time on private life; Providing informal care; Partner isretired; More time needed for activities outside work; Enjoy life as long as health permits; Morefreedom, less obligations

M + includes: Enough challenges at work; Social contacts at work

L includes: Request of employer or colleague; Partner is still working

^a^In 2019, only one reason could be given. In 2022, more than one reason could be given; thepercentages for 2022 are recalculated to add up to 100%

Roughly one-half of continuing workers (49%) wished to retire before the SPA if this would be made financially possible. They were in statistically significantly poorer health than workers who wished to continue working regarding functional limitations, self-rated health, and depressive symptoms, whereas their cognitive ability was not different (Supplement Table 4). A large share gave high work demands or health limitations as their reason for wishing to retire. A few were no longer motivated to work, amongst others because of organisational changes at the workplace. The majority, meanwhile, gave as a reason the wish to have more time for their private life, and a minority expected financial benefit (Table 3, second panel). Among these reasons, the reason ‘work demands and health’ was significantly associated with functional limitations, less-than-good self-rated health, and depressive symptoms. A lack of motivation was inversely associated with less-than-good self-rated health. No other motivation-health associations were statistically significant (Supplement Table 4).

Among the other half of continuing workers, wishing to continue working even if retirement before the SPA would be made financially possible, motivations were predominantly related to positive aspects of the job: enough challenges and contacts with colleagues. Only a few gave other reasons, such as loyalty to others, e.g. when their partner was still working, and financial benefit (Table 3, third panel). No significant associations with health emerged (Supplement Table 4).

#### Hours worked

Participants who continued working did so at approximately the same number of hours a week in periods 1 and 2, but had substantially reduced their working hours in period 3. This drop proved significant in a multivariable Generalised Estimating Equation model.

**Fig. 1:**
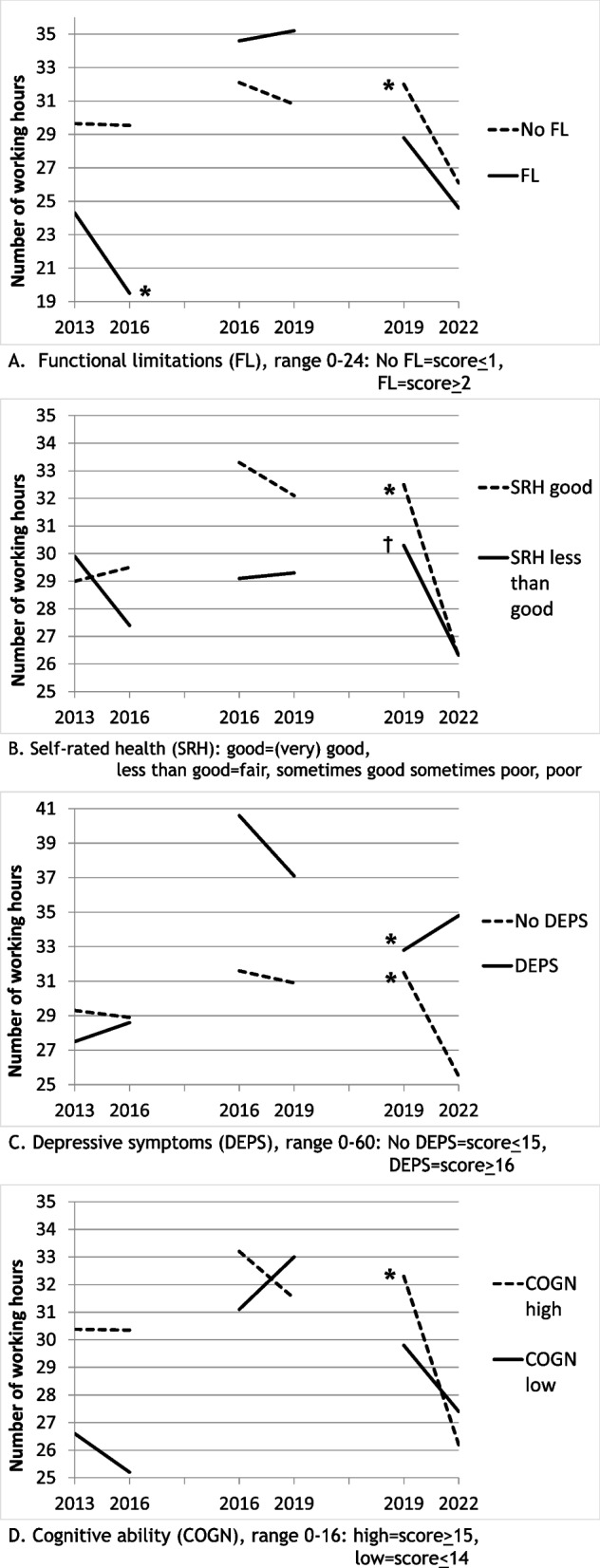
Longitudinal change in working hours in workers aged 61–63 years at baseline to 64-SPA at follow-up: association with functional limitations (Panel **A**), self-rated health (Panel **B**), depressive symptoms (Panel **C**), and cognitive ability (Panel **D**). Estimated marginal means from General Estimating Equations, adjusted for age, sex, educational level, and partner status. * Decrease is significant at *p* < 0.05; ^**†**^ decrease is significant at *p* < 0.10. Note: for parameter values see Table 3

The health indicators affected change in working hours in different ways (Table 4 and Fig. 1). A significant decrease in working hours was found among workers with functional limitations only in period 1; the coefficient of the interaction functional limitations*within-person time significantly deviated from that in later periods (Table 4, first panel, third line). By contrast in period 3, working hours decreased regardless of the existence of functional limitations, as the interaction functional limitations*time was non-significant (Table 4, first panel, first and third lines). Likewise, less-than-good self-rated health negatively affected working hours only in period 1, although the decrease in working hours did not reach significance (Table 4, second panel, third line); in period 3, working hours decreased significantly regardless of self-rated health (Table 4, second panel, first and third lines). Depressive symptoms show a different picture (Table 4, third panel, first and third line): in periods 1 and 2, change in hours worked did not differ between workers without and with depressive symptoms. In period 3, however, workers without depressive symptoms significantly reduced their working hours, whereas workers with depressive symptoms did not. Workers with high and low cognitive ability did not significantly differ regarding change in working hours in periods 1 and 2, but in period 3, workers with high cognitive ability significantly decreased their working hours and this change was significantly greater than in previous periods (Table 4, fourth panel, first and third line). Workers with low cognitive ability showed a smaller, non-significant decrease in working hours in period 3.

**Table 4:** Associations of four health indicators with 3-year change in working hours in continuing-workers aged 61–63 years at baseline, for three periods. Linear Generalised Estimating Equations^a^ (N(observations) = 444)

	Period 1: 2013–2016		Period 2: 2016–2019		Period 3: 2019–2022	
	B	95% CI	B	95% CI	B	95% CI
*Functional limitations, dichotomous: 0* = *scores 0 or 1; 1* = *scores 2 or higher*						
Change (within-person time)	−0.11^b^	−3.16; 2.94	−1.26^b^	−4.24; 1.73	−5.92^c^	−8.56; −3.29**
Functional limitations	−5.38	−13.78; 3.01	2.56	−3.42; 8.53	−3.18	−8.10; 1.73
Time*Functional limitations	−4.62^d^	−8.85; −0.38*	1.80^e^	−2.99; 6.59	1.93^e^	−4.99; 8.85
*Self-rated health, dichotomous: 0* = *(very) good; 1* = *less than good*						
Change (within-person time)	0.53^b^	−2.93; 3.99	−1.17^b^	−4.23; 1.89	−6.26^c^	−9.17; −3.34**
Self-rated health	0.88	−4.82; 6.58	−4.13	−10.33; 2.08	−2.24	−6.80; 2.32
Time*Self-rated health	−2.94^d^	−9.07; 3.20	1.34	−2.87; 5.55	2.31	−2.72; 7.35
*Depressive symptoms, dichotomous: 0* = *scores 0–15; 1* = *scores 16 or higher*						
Change (within-person time)	−0.42^b^	−3.56; 2.72	−0.74^b^	−3.45; 1.97	−5.99^c^	−8.54; −3.44**
Depressive symptoms	−1.80	−14.73; 11.14	8.96	−2.18; 20.10	1.30	−3.32; 5.91
Time* Depressive symptoms	1.50^b^	−2.85; 5.85	−2.75^b^	−11.78; 6.28	7.97^c^	4.91; 11.03**
*Cognitive ability, dichotomous: 0* = *scores 15 or 16; 1* = *scores 14 or lower*						
Change (within-person time)	−0.03^b^	−3.62; 3.56	−1.67^b^	−4.06; 0.72	−6.07^c^	−8.57; −3.57**
Cognitive ability	−3.81	−11.57; 3.95	−2.12	−9.90; 5.66	−2.46	−10.19; 5.28
Time*Cognitive ability	−1.31	−6.21; 3.59	3.63^e^	−5.60; 12.86	3.62^e^	−7.97; 15.21

^a^Adjusted for age, sex, educational level, and partner status

^b^Coefficients in periods 1 and 2 are significantly different from coefficient in period 3

^c^Coefficient in period 3 is significantly different from coefficients in periods 1 and 2

^d^Coefficient in period 1 is significantly different from coefficients in periods 2 and 3

^e^Coefficients in periods 2 and 3 are significantly different from coefficient in period 1

^†^*p* < 0.10; * *p* < 0.05; ** *p* < 0.001

Motivations to continue working or not, if made financially possible, were not significantly different for workers who reduced and did not reduce their working hours by 4 or more hours (Supplement Table 5). There was one notable exception: among workers who reduced their working hours, a larger share reported a lack of motivation to work than among workers who did not.

## Discussion

Our study addressed late-career workers’ changes in work participation up to state pension age (SPA) and the role of health and motivation therein during 2013–2022, a period of quickly rising SPA. During the study period, our data showed that a rising proportion of workers remained in the workforce. This was particularly so from 2016 to 2019. The non-linearity of this rise is reflected in the proportions involved in paid work based on the full Dutch population [50] (Supplement Fig. 1). This finding may have two tentative explanations. First, in the period 2013–2015, the rise in SPA amounted to only three months, so that little rise could be expected. Second, the period after 2019 was dominated by the COVID-19 pandemic. In this period, we also observed a decrease in hours worked, which was also reflected in data on the full Dutch population, where we see a decrease in full-time (i.e., 36 + hours) work participation from 2020 onwards [50] (Supplement Fig. 2). The COVID-19-related containment measures involved changes in the organisation of work for many workers; for example, working from home proliferated quickly [37]. Changes in the organisation of work may have led workers to retire earlier than originally planned [40]. Also, the slower pace of life related to the social restrictions may have led people to think about what is really important in life [39]. Our data on motivation to work show a substantial share of lack of motivation to work among workers who retired from the workforce and among workers who reduced their working hours between 2019 and 2022. These findings suggest that for many workers in the age group studied, work emerged as less important than other aspects of life during the COVID-19 pandemic.

According to population data available up to 2023, the lower number of working hours in the age group studied persisted through 2023 [50]. This may be related to after-effects of the COVID-19 pandemic measures. For example, more late-career workers may have used the option for part-time pension to which employees have a legal right [51]. A practical consequence is that workforce participation does not increase as quickly as expected when only continuation of work is considered. Ultimately, this may have consequences for policy measures such as the rate at which the SPA is raised in the future.

In our late-career age group, health was generally neither associated with transition out of the workforce nor with hours worked. This finding ties in with reports on increases in working life expectancy in poor health over the past decades, suggesting that the importance of health for work ability has decreased [30–32]. This may reflect societal developments such as the increase in educational level and the decrease of physical labour which enable late-career workers to work until older ages. It may also reflect an effect noted by Mortelmans & Vannieuwenhuyze, i.e., as workers approach SPA the effect of health on retirement becomes weaker [52]. Vice versa, workers with work-limiting health problems may have left the workforce at younger ages: a healthy worker effect. This is supported by our finding of poorer scores on all four health indicators of non-workers compared to workers (Table 1). Workers approaching SPA may feel a greater financial necessity to continue working. They may hide their health problems for their employer for fear of losing their job [53]. On the positive side, a positive work commitment has been shown to decrease workforce exit through disability pension [26], and thus continuing workers may have a greater commitment to work and greater work satisfaction, which compensates for experienced health problems [20, 23]. Alternatively, they may have developed coping strategies to deal with health problems or they may have been able to make work adjustments according to their needs, thereby attenuating the negative effect of poor health on workforce participation [12, 54]. Moreover, company measures to manage the ageing workforce, including work adjustments, may have become more effective [55]. Another reason why we did not find an association between health and subsequent work state may be that we studied exit from the workforce up to statutory retirement age. The association is more likely to hold for workers exiting earlier. However, sensitivity analyses examining this association excluding respondents who exited less than one half year before their SPA, yielded similar results (Supplementary Table 6).

There were two exceptions to the lack of health associations. First, a poorer self-rated health predicted exit from the workforce in the period 2019–2022, but not in earlier periods. Earlier studies, that generally included workers with younger minimum ages, present ample evidence that poor self-rated health predicts exit from the workforce among late-career workers [8, 9]. Van den Berg and colleagues showed that self-rated health was a stronger predictor of workforce exit than functional limitations [56]. These authors explain this finding by noting that self-perceived health includes both physical and mental health, whereas functional limitations primarily concern physical health. In our study, neither functional limitations nor specific mental health measures alone predicted exit. It seems therefore well possible that a comprehensive measure such as self-rated health captures enough health aspects to be able to predict workforce exit.

An explanation for our finding that self-rated health predicted workforce exit only in the last period, in which SPA was highest, is not self-evident. Not only was SPA highest, but also the COVID-19 pandemic took place in this period. In the context of the LASA-study, two questionnaires sent out to LASA-participants during the pandemic enabled us to examine the influence of the pandemic on work participation (Supplementary Table 7). Self-rated health turned out to be associated with subsequent workforce exit mostly before the start of the pandemic, which excludes the pandemic as a major explanation. A more likely explanation may be the higher age and the longer exposure to work demands of late-career workers in the period 2019–2022, both of which are associated with slower recovery from work strain [15, 57], which in turn affects self-rated health [58]. This likely greater role of health as workers need to continue up to higher SPA is a cause of concern for the feasibility of extending working lives.

As a second exception to the general finding that health was not associated with workforce participation, functional limitations and poor self-rated health predicted a decrease in hours worked only in the first period. This finding corresponds to earlier evidence [11]. However, we found no association of physical health with hours worked in the subsequent periods, which supports our second expectation that the role of health decreases over time. In the third period we even found that better mental health, i.e., fewer depressive symptoms and better cognitive ability, were predictive of a greater decrease in hours worked. Workers with depressive symptoms have been shown to benefit from participation in the workforce, because it provides structure to their day [59]. However, we also found that depressed workers reported issues with health and work demands as a reason to wish to retire early if this would be made financially possible. These workers would be better facilitated to continue working by closely monitoring the fit between their work ability and their work demands [60]. Furthermore, workers with better cognitive ability are likely to have cognitively demanding jobs, which may compromise mental health [35, 36]. These workers are also likely to have better salaried jobs, which may provide them with enough financial resources to afford working fewer hours.

Motives for retirement from the workforce consisted mostly of the greater importance attached to private life. Other motives were issues with work demands and health and lack of motivation to work. Notably, the latter reason was reported only in 2022 and was associated with better self-rated health. Therefore, the reported lack of motivation to work may have had its origin in the changes in work organisation during the COVID-19 pandemic [40].

Motivation to continue working centred around positive aspects of work, while smaller minorities reported loyalty to others and financial benefit. Wishes to stop working if a financial possibility would arise, were motivated in approximately equal shares by issues with work demands and health and the lack of motivation to work on the one hand, and the greater importance attached to private life as well as financial benefit on the other hand. Continuing workers wishing to stop working reported poorer health and more depressive symptoms than their counterparts who in this case wished to continue working. This finding supports our earlier suggestion that late-career workers may feel a greater financial necessity to continue working, despite health problems.

Our findings provide some support for the extensive earlier evidence on the importance of good health for extending working life [8, 9]. However, good health may be less important in the age-group studied than in somewhat younger age-groups. Instead, our findings highlight the importance of motivation to work, which proved to be particularly prominent during the COVID-19 pandemic. Thus, while not ignoring the importance of the maintenance of a good match between work demands and health, sufficient attention to motivation to work in late-career workers seems imperative. Working conditions such as autonomy and task variation, but also work quality including having self-crediting and meaningful tasks, using one’s experiences and knowledge, and receiving recognition for one’s work may help late-career workers to maintain sufficient motivation to work. In addition, the opportunity to develop and use new skills may help in maintaining motivation to work [23, 27, 61].

### Strengths and limitations

Our study has several unique aspects. First, our focus is on an age group of late-career workers in which only very recently workforce participation has substantially increased. This age group is usually studied as part of a wider age group, such as 55–64 years or even 45–64 years, so that its unique experience is not visible. Second, our study addresses workforce participation over time. Whereas much research is done on changes in workforce participation in relation to policy measures to extend working lives per se as well as on the predictive ability of health for workforce participation, to the best of our knowledge no studies have addressed changes in the predictive ability of health for workforce participation over time. Third, whereas many studies use only one or a few health indicators, our study uses four indicators, both subjective and objective, and covering physical and mental as well as cognitive health. Fourth, the inclusion of motivation facilitates the interpretation of our findings regarding health.

An obvious limitation of our study is its small sample size. It prohibited us from investigating predictors of specific exit-routes as well as re-employment across the three periods studied; in previous studies, the share of each exit route and re-entrance into the workforce among older non-workers has been shown to be small [10, 62, 63]. Also, we were not able to distinguish specific groups, such as based on gender and educational level; differences between these groups can be substantial regarding associations of health, motivation, and workforce participation. Furthermore, the relatively small power may prohibit the emergence of statistically significant findings. Regardless, the findings that do show statistical significance represent substantial effects. Replication in larger studies is recommendable.

A second limitation is that our design includes for each participant only two waves three years apart. It would be preferable to have more waves closer together, allowing the estimation of shorter-term fluctuations in both health and workforce participation [10].

Third, although we distinguished two key indicators of workforce participation, based on the time spent on paid work (be it zero hours as in exit from the workforce or the number of hours worked) also sickness absence reduces hours worked and can be considered to be an aspect of sustainable working life [64]. Unfortunately, data on sickness absence were not available throughout the period studied.

A fourth limitation concerns the availability of data on motivation. In the 2019 wave, the first wave that motivation was measured, participants were allowed to indicate only their main motive. This was redressed in the 2022 wave, when multiple motives could be indicated. Here, among the participants who would wish to continue working, the majority gave one motive – this motive being positive aspects of work. By contrast, among the participants who exited or who would wish to exit, the majority gave more than one motive, in accordance with earlier, qualitative research [24]. Moreover, a lack motivation to work was reported mostly when other motives were reported, which indicates that lack of motivation was not considered to be a main motive, or in any case was not generally reported as such.

Finally, although the response categories included a broad range of motives and an additional response category ‘other’ allowed participants to provide a motive in their own words, potential other motivational factors were not measured. Various conceptualisations of motivation to work have been developed. Examples are meaningfulness, commitment, and engagement, together constituting intrinsic motivation [29], self-regulation processes of work engagement or disengagement [65–67], work centrality as derived from social identity theory [68], and autonomous or intrinsic versus controlled or extrinsic motivation based on self-determination theory [69]. Different definitions of work motivation likely connect to different work-related outcomes. Future research may address how motivational factors in other conceptualisations evolve over time in late-career workers, thus providing directions for motivational interventions.

### Conclusions and implications

We examined workforce participation from 2013 to 2022, a period of gradually rising state pension age from a health and motivational perspective. We found that at ages 61 to SPA, the proportion of workers that remained in the workforce rose substantially up to 2019 and declined somewhat after that. Health played a minor role in transitions out of the workforce and in changes in hours worked. Possibly, financial need overrode the role of health, given the finding that functional limitations, self-rated health, and depressive symptoms correlated with the wish to stop working earlier if the financial situation would allow it. Only in the period 2019 up to the COVID-19 outbreak, self-rated health predicted exit from the workforce, and only in the earliest period, 2013–2016, physical health predicted a decrease in hours worked. During the COVID-19 pandemic, changes in the organisation of much work may have led to a lower work satisfaction and the slowing down of life in general may have decreased the motivation to work. This was apparent from the substantial decrease in working hours, a decrease that was not associated with poor health, and from the more frequently reported lack of motivation to work in this period. Whether the lower workforce participation during the COVID-19 pandemic was temporary remains for future research.

Whereas measures to accommodate health problems of older workers are relatively common by now [54], effective interventions to enhance work motivation have been developed more recently. One example is a randomised controlled trial addressing older employees’ preparedness and self-efficacy to adapt to a new career phase, manage career barriers, take advantage of resources, and protect and enhance their own career. This trial showed positive effects on employees’ work engagement and motivation to continue their working careers [70].

Extension of working life is common in most Western countries, amongst others due to increases in SPA [71]. Although our study is limited to the Netherlands, this country has seen one of the fastest rises in late-career employment rates in Europe [2]. Thus, our findings are likely to apply to other countries that are raising or plan to raise SPA. Our findings suggest that the feasibility of extending working life and thus of maintaining late-career workers in the workforce, not only requires attention to health maintenance, but also to factors that promote the motivation to work.

## Supplementary Information

Supplementary Material 1.

## Data Availability

The dataset generated and analysed is available for replication purposes, provided an agreement is made up. Please see (http://www.lasa-vu.nl).

## Abbreviations

CI: Confidence Interval
GEE: Generalised Estimating Equations
LASA: Longitudinal Aging Study Amsterdam
OR: Odds Ratio
SPA: State Pension Age

## Footnotes

1 For readability, we denote each wave by the second year of data collection, because most participants were interviewed in this year.

## References

[1] OECD. Design of pension systems. In: Pensions at a glance 2023. Paris, France: Organisation of Economic Co-operation and Development; 2023.

[2] Henkens K, van Solinge H, Damman M, Dingemans E. Taken by surprise: how older workers struggle with a higher retirement age. Demos. 2016;32(7):1–2.

[3] Eurostat. Employment rates – annual statistics. Statistics explained, updated 12/06/2025. https://ec.europa.eu/eurostat/databrowser/view/lfsa_ergacob_custom_17789431/default/table.

[4] Euwals R. The labour market for older workers: mechanisms and institutions. Economist. 2014;162:309–13. 10.1007/s10645-014-9243-7.

[5] Montizaan R, Cobben L. Vijfde editie van de AOW Monitor 2023: effect van verhoging van de AOW-leeftijd op werk, inkomen en gezondheid [Fifth edition of the SPA Monitor 2023: effect of raising the SPA on work, income, and health]. ROA Report ROA-R-2024/1. Maastricht: Researchcentrum voor Onderwijs en Arbeidsmarkt;2024.

[6] Rabaté S, Rochut J. Employment and substitution effects of raising the statutory retirement age in France. J Pension Econ Fin. 2020;19(3):293–308. 10.1017/S1474747218000392.

[7] Nusselder W, Rado MK, Deeg DJH. Arbeidsmarktstatus tussen de 65ste verjaardag en de AOW-leeftijd: verschillen tussen opleidingsgroepen [Labour market status between the 65th birthday and the statutory retirement age: differences between educational groups]. Netspar Design Paper 199. Tilburg: Netspar 2021.

[8] van Rijn RM, Robroek SJW, Brouwer S, Burdorf A. Influence of poor health on exit from paid employment: a systematic review. Occup Environ Med. 2014;71:295–301. 10.1136/oemed-2013-101591. 24169931 10.1136/oemed-2013-101591

[9] de Breij S, Mäcken J, Qvist JY, Holman D, Hess M, Huisman M, et al. Educational differences in the influence of health on early work exit among older workers. Occup Environ Med. 2020;77(8):568–75. 10.1136/oemed-2019-106253. 32269132 10.1136/oemed-2019-106253 PMC7402445

[10] van de Ven D, Robroek SJW, Oude Hengel KM, Burdorf A, Schuring M. Changes in health among 45–64-year-old Dutch persons before, during and after becoming unemployed or employed: a seven year follow-up study. Scand J Work Environ Health. 2022;48(4):283–92. 10.5271/sjweh.4016. 35260909 10.5271/sjweh.4016 PMC9524163

[11] Flores M, Kalwij A. What do wages add to the health-employment nexus? Evidence from older European workers. Oxf Bull Econ Stat. 2019. 10.1111/obes.12257.

[12] Pransky GS, Benjamin KL, Savageau JA, Currivan D, Fletcher K. Outcomes in workrelated injuries: a comparison of older and younger workers. Am J Ind Med. 2005;47:104–12. 10.1002/ajim.20122. 15662646 10.1002/ajim.20122

[13] Feer S, Lipps O, Dratva J, Baumann I. Health and labor force participation among older workers in Switzerland: a growth curve analysis. Eur J Ageing. 2022;19:1395–406. 10.1007/s10433-022-00716-z. 36506689 10.1007/s10433-022-00716-z PMC9729446

[14] Schuring M, Burdorf L, Kunst A, Mackenbach J. The effects of ill health on entering and maintaining paid employment: evidence in European countries. J Epidemiol Community Health. 2007;61:597–604. 10.1136/jech.2006.047456. 17568051 10.1136/jech.2006.047456 PMC2465755

[15] van der Noordt M, van Tilburg TG, van der Pas S, Wouterse B, Deeg DJH. Health trajectories across the work exit transition in the 1990s, 2000s, and 2010s: the role of working conditions and policy. Arch Public Health. 2023;81:16. 10.1186/s13690-022-01008-9. 36740687 10.1186/s13690-022-01008-9 PMC9901107

[16] Spitz-Oener A. Technical change, job tasks, and rising educational demands: looking outside the wage structure. J Labor Econ. 2006;24(2):235–270. 0734-306X/2006/2402-0002$10.00.

[17] Ciliacus R, Hijdra RW, Robroek SJW, Leist AK, Burdorf A, Schuring M. Memory function and early exit from paid employment through different pathways among ageing European workers. Scand J Work Environ Health. 2025;51(2):89–99. 10.5271/sjweh.4211. 39903760 10.5271/sjweh.4211 PMC11893212

[18] Ihle A, Borella E, Rahnfeld M, Müller SR, Enge S, Hacker W, et al. The role of cognitive resources for subjective work ability and health in nursing. Eur J Ageing. 2015;12:131–40. 10.1007/s10433-014-0331-y. 28804351 10.1007/s10433-014-0331-y PMC5549137

[19] Roy SB. Effect of health on retirement of older Americans: a competing risks study. J Labor Res. 2018;39(1):56–98. 10.1007/s12122-017-9255-6.

[20] Hasselhorn HM, Leinonen T, Bültmann U, Mehlum IS, du Prel J-B, Kiran S, et al. The differentiated roles of health in the transition from work to retirement – conceptual and methodological challenges and avenues for future research. Scand J Work Environ Health. 2022;48(4):312–21. 10.5271/sjweh.4017. 35239972 10.5271/sjweh.4017 PMC9524164

[21] de Wind A, Geuskens GA, Ybema JF, Bongers PM, van der Beek AJ. The role of ability, motivation, and opportunity to work in the transition from work to early retirement – testing and optimizing the early retirement model. Scand J Work Environ Health. 2015;41(1):24–35. 10.5271/sjweh.3468. 25393088 10.5271/sjweh.3468

[22] Kanfer R, Beier ME, Ackerman PL. Goals and motivation related to work in later adulthood: an organizing framework. Eur J Work Organ Psy. 2013;22(3):253–64. 10.1080/1359432X.2012.734298.

[23] Nilsson K. Conceptualisation of ageing in relation to factors of importance for extending working life – a review. Scand J Public Health. 2016;44:490–505. 10.1177/1403494816636265. 26976390 10.1177/1403494816636265

[24] Reeuwijk KG, de Wind A, Westerman MJ, Ybema JF, van der Beek AJ, Geuskens GA. ‘All those things together made me retire’: qualitative study on early retirement among Dutch employees. BMC Public Health. 2013;13:516. 10.1186/1471-2458-13-516. 23714371 10.1186/1471-2458-13-516 PMC3674915

[25] Meng A, Sundstrup E, Andersen LL. Factors contributing to retirement decisions in Denmark: comparing employees who expect to retire before, at, and after the state pension age. Int J Environ Res Public Health. 2020;17:3338. 10.3390/ijerph17093338. 32403380 10.3390/ijerph17093338 PMC7246757

[26] Clausen T, Burr H, Borg V. Does affective organizational commitment and experience of meaning at work predict risk of disability pensioning? An analysis of register-based outcomes using pooled data on 40,554 observations in four occupational groups. Am J Ind Med. 2014;57:709–17. 10.1002/ajim.22313. 24619706 10.1002/ajim.22313

[27] Garthe N, Hasselhorn HM. Wollen und können ältere Beschäftigte länger erwerbstätig bleiben, wenn sich ihre Arbeit verbbessert? [Is improvement of working conditions related to an increase in the subjective employment perspective of older workers?]. Zentralbl Arb-ArbErgon. 2023;73(2):49–63.

[28] Allan BA, Dexter C, Kinsey R, Parker S. Meaningful work and mental health: job satisfaction as a moderator. J Ment Health. 2018;27(1):38–44. 10.1080/09638237.2016.1244718. 27841056 10.1080/09638237.2016.1244718

[29] Chalofsky N, Krishna V. Meaningfulness, commitment, and engagement: the intersection of a deeper level of intrinsic motivation. Adv Dev Hum Resour. 2009;11(2):189–203. 10.1177/1523422309333147.

[30] de Wind A, van der Noordt M, Deeg DJH, Boot CRL. Working life expectancy in good and poor selfperceived health among Dutch workers aged 55–65 years with a chronic disease over the period 1992–2016. Occup Environ Med. 2018;75(11):792–7. 10.1136/oemed-2018-105243. 30194272 10.1136/oemed-2018-105243

[31] van der Noordt M, van der Pas S, van Tilburg TG, van den Hout A, Deeg DJH. Changes in working life expectancy with disability in the Netherlands, 1992–2016. Scand J Work Environ Health. 2019;45(1):73–80. 10.5271/sjweh.3765. 30176168 10.5271/sjweh.3765

[32] Beller J, Sperlich S, Epping J, Tetzlaff J. Trends in severe functional limitations among working and non-working adults in Germany: towards an (un)-healthy working life? Eur J Ageing. 2024;21:13. 10.1007/s10433-024-00809-x. 38652375 10.1007/s10433-024-00809-x PMC11039435

[33] Amilon A, Larsen M. Increasing retirement ages in Denmark: do changes in gender, education, employment status and health matter? Eur J Ageing. 2023;20:24. 10.1007/s10433-023-00771-0. 37329473 10.1007/s10433-023-00771-0 PMC10276799

[34] Romeu Gordo L, Skirbekk V. Skill demand and the comparative advantage of age: Jobs tasks and earnings from the 1980s to the 2000s in Germany. Labour Econ. 2013;22:61–9. 10.1016/j.labeco.2012.09.003.

[35] Borle P, Boerner-Zobel F, Voelter-Mahlknecht S, Hasselhorn HM, Ebener M. The social and health implications of digital work intensification. Associations between exposure to information and communication technologies, health and work ability in different socio-economic strata. Int Arch Occup Environ Health. 2021;94:377–90. 10.1007/s00420-020-01588-5. 33084928 10.1007/s00420-020-01588-5 PMC8032606

[36] Green F, Felstead A, Gallie D, Henseke G. Working still harder. ILR Rev. 2022;75(2):458–87. 10.1177/0019793920977850.

[37] Huls SPI, Sajjad A, Kanters TA, Hakkaart-van Roijen L, Brouwer WBF, van Exel J. Productivity of working at home and time allocation between paid work, unpaid work and leisure activities during a pandemic. Pharmacoeconomics. 2022;40:77–90. 10.1007/s40273-021-01078-7. 34472047 10.1007/s40273-021-01078-7 PMC8410454

[38] Davis OF, Quinby LD, Rutledge MS, Wettstein G. How did COVID-19 affect the labor force participation of older workers in the first year of the pandemic? J Pension Econ Fin. 2023;22:509–523. 1017/S1474747223000045.

[39] Renckens SC, Pasman HR, Huisman M, Hoogendijk EO, Onwuteaka-Philipsen BD. Self-reported changes in personal development and meaning in life among older adults during the COVID-19 pandemic: results from the Longitudinal Aging Study Amsterdam. Health Soc Care Community. 2023;9969216. 10.1155/2023/9969216.

[40] D’Angelo S, Bloom I, Ntani G, Walker-Bone K. Why did middle-aged and older people retire since the first COVID-19 lockdown? A qualitative study of participants from the Health and Employment After Fifty study. BMC Public Health. 2024;24:103. 10.1186/s12889-023-17548-w. 38183033 10.1186/s12889-023-17548-w PMC10770915

[41] Hoogendijk EO, Deeg DJH, de Breij S, Klokgieters SS, Kok AAL, Stringa N, et al. The longitudinal aging study Amsterdam: cohort update 2019 and additional data collections. Eur J Epidemiol. 2020;35(1):61–74. 10.1007/s10654-019-00541-2. 31346890 10.1007/s10654-019-00541-2 PMC7058575

[42] McWhinnie JR. Disability assessment in population surveys: results of the O.E.C.D. Common Development Effort. Rev Epidemiol Sante. 1981;29(4):413–419. 6461907

[43] Radloff LS. The CES-D scale: a self-report depression scale for research in the general population. Appl Psychol Meas. 1977;1:385–401.

[44] Beekman ATF, Deeg DJH, van Limbeek J, Braam AW, de Vries MZ, van Tilburg W. Criterion validity of the center for epidemiologic studies depression scale (CES-D): results from a community based sample of older subjects in the Netherlands. Psychol Med. 1997;27:231–5. 10.1017/s0033291796003510. 9122304 10.1017/s0033291796003510

[45] Folstein MF, Folstein SE, McHugh PR. Mini-mental state: a practical method for the clinician. J Psychiatr Res. 1975;12(3):189–98. 10.1016/0022-3956(75)90026-6. 1202204 10.1016/0022-3956(75)90026-6

[46] Gardeniers MKM, Broese van Groenou MI, Meijboom EJ, Huisman M. Three-year trajectories in functional limitations and cognitive decline among Dutch 75+ year olds, using ninemonth intervals. BMC Geriatr. 2022;22:89. 10.1186/s12877-021-02720-x. 10.1186/s12877-021-02720-x PMC8805337 35105338

[47] Proper KI, Deeg DJH, Van der Beek AJ. Challenges at work and financial rewards to stimulate longer workforce participation. Hum Resour Health. 2009;11(7):70. 10.1186/1478-4491-7-70 PMC2731068 19671142

[48] Figueiras A, Domenech-Massons JM, Cadarso C. Regression models: calculating the confidence interval of effects in the presence of interactions. Stat Med. 1998;17:2099–105. 10.1002/(SICI)1097-0258(19980930)17:18%3c2099::AID-SIM905%3e3.0.CO;2-6. 9789916 10.1002/(sici)1097-0258(19980930)17:18<2099::aid-sim905>3.0.co;2-6

[49] Aiken L, West S, Reno R. Multiple regression: testing and interpreting interactions. Newbury Park, CA: Sage Publications; 1991.

[50] Statistics Netherlands. Work participation and unemployment in older people. www.opendata.nl/statline. Accessed 26 November 2024.

[51] Bolhaar J, van Vuuren D. Geleidelijke uittreding en de rol van deeltijdpensioen [Gradual retirement and the role of part-time pensions]. Netspar Design Paper 100. Tilburg: Netspar; 2018.

[52] Mortelmans D, Vannieuwenhuyze JTA. The age-dependent influence of self-reported health and job characteristics on retirement. Int J Public Health. 2013;58:13–22. 10.1007/s00038-012-0411-8. 23007875 10.1007/s00038-012-0411-8 PMC3557381

[53] Lain D, van der Horst M, Vickerstaff S. Adapting to an older workforce: health and the (non) response of employers in an era of insecurity. J Soc Policy. 2024 Early Access. 10.1017/S0047279424000163.

[54] Nemoto Y, Takahashi T, Nonaka K, Hasebe M, Koike T, Minami U, et al. Working for only financial reasons attenuates the health effects of working beyond retirement age: a 2-year longitudinal study. Geriatr Gerontol Int. 2020;20:745–51. 10.1111/ggi.13941. 32618090 10.1111/ggi.13941

[55] Turek K, Oude Mulders J, Henkens K. The proactive shift in managing an older workforce 2009–2017: a latent class analysis of organizational policies. Gerontologist. 2020;60(8):1515–26. 10.1093/geront/gnaa037. 32364231 10.1093/geront/gnaa037 PMC7681210

[56] van den Berg TIJ, Elders LAM, Burdorf A. Influence of health and work on early retirement. J Occup Environ Med. 2010;52:576–83. 10.1097/JOM.0b013e3181de8133. 20523241 10.1097/JOM.0b013e3181de8133

[57] Kiss P, De Meester M, Braeckman L. Differences between younger and older workers in the need for recovery after work. Int Arch Occup Environ Health. 2008;81(3):311–20. 10.1007/s00420-007-0215-y. 17576592 10.1007/s00420-007-0215-y

[58] Headrick L, Newman DA, Park YA, Liang Y. Recovery experiences for work and health outcomes: a meta-analysis and recovery-engagement-exhaustion model. J Bus Psychol. 2023;38:821–64. 10.1007/s10869-022-09821-3.

[59] Boot CR, de Kruif AT, Shaw WS, van der Beek AJ, Deeg DJ, Abma T. Factors important for work participation among older workers with depression, cardiovascular disease, and osteoarthritis: a mixed method study. J Occup Rehabil. 2016;26(2):160–72. 10.1007/s10926-015-9597-y. 26210996 10.1007/s10926-015-9597-y PMC4854935

[60] Boot CRL, van den Heuvel SG, Bültmann U, de Boer AGEM, Koppes LLJ, van der Beek AJ. Work adjustments in a representative sample of employees with a chronic disease in the Netherlands. J Occup Rehabil. 2013;23:200–8. 10.1007/s10926-013-9444-y. 23592014 10.1007/s10926-013-9444-y

[61] El Khawli E, Visser M, Firat M. Trajectories of job resources and the timing of retirement. Work Aging Retire. 2025;11:149–61. 10.1093/workar/waae004. 40151215 10.1093/workar/waae004 PMC11937890

[62] Atav T, Jongen E, Rabaté S. The effects of the increase in the retirement age in the Netherlands. CPB Discussion Paper. The Hague: Netherlands Central Planning Office 2019.

[63] Chan S, Stevens AH. Job loss and employment patterns of older workers. J Labor Econ. 2001;19(2):484–521. 0734-306X/2001/1902-0008

[64] Ropponen A, Wang M, Narusyte J, Silventoinen K, Böckerman P, Svedberg P. Sustainable working life in a Swedish twin cohort – a definition paper with sample overview. Int J Environ Res Public Health. 2021;18:5817. 10.3390/ijerph18115817. 34071494 10.3390/ijerph18115817 PMC8197988

[65] Kooij D, Zacher H, Wang M, Heckhausen J. Successful aging at work: a process model to guide future research and practice. Ind Organ Psychol. 2020;13(3):345–65. 10.1017/iop.2020.1.

[66] Damman M, Henkens K, Kalmijn M. Late-career work disengagement: the role of proximity to retirement and career experiences. J Gerontol B Psychol Sci Soc Sci. 2013;68(3):455–63. 10.1093/geronb/gbt001. 23407785 10.1093/geronb/gbt001

[67] de Wind A, Leijten FRM, Hoekstra T, Geuskens GA, Burdorf A, van der Beek AJ. “Mental retirement?” Trajectories of work engagement preceding retirement among older workers. Scand J Work Environ Health. 2017;43(1):34–41. 10.5271/sjweh.3604. 27907223 10.5271/sjweh.3604

[68] Macdonald J, Levy SL. Ageism in the workplace: the role of psychosocial factors in predicting job satisfaction, commitment, and engagement. J Soc Issues. 2016;72(1):169–90. 10.1111/josi.12161.

[69] Henning G, Stenling A, Tafvelin S, Hansson I, Kivi M, Johansson B, et al. Preretirement work motivation and subsequent retirement adjustment: a self-determination theory perspective. Work Aging Retire. 2019;5(2):189–203. 10.1093/workar/way017.

[70] Vuori J, Törnroos K, Ruokolainen M, Wallin M. Enhancing late-career management among aging employees – a randomized controlled trial. J Vocational Behav. 2019;115:103327. 10.1016/j.jvb.2019.103327.

[71] Turek K, Henkens K, Kalmijn M. Gender and educational inequalities in extending working lives: late-life employment trajectories across three decades in seven countries. Work Aging Retire. 2024;10:100–22. 10.1093/workar/waac021.

