# Supplementary material for: Late-career workforce participation in times of rising state pension age: the role of health and motivation
Source: BMC Public Health. 2025 Nov 21;25:4404. doi: 10.1186/s12889-025-25556-1 (PMC12754992; doi:10.1186/s12889-025-25556-1)
Supplement: Supplementary file 1 — Supplementary Material 1. [file 12889_2025_25556_MOESM1_ESM.docx]

**Supplementary material**

**Late-career workforce participation in times of rising statutory retirement age: the role of health and motivation**

**Supplement Table 1.** Drop-out during 3-year follow-up among workers aged 61-63 years at baseline: bivariate association with baseline characteristics across three periods (Odds Ratios, 95% Confidence Intervals).

|  | OR | 95% CI |
| --- | --- | --- |
| Period: |  |  |
| - Period 2 vs period 1 | 0.45 | 0.21-0.95* |
| - Period 3 vs period 1 | 0.42 | 0.20-0.90* |
| Age per year | 0.70 | 0.45-1.08 |
| Sex: female vs male | 0.97 | 0.52-1.81 |
| Education: |  |  |
| - Intermediate vs low | 1.12 | 0.51-2.45 |
| - High vs low | 0.70 | 0.30-1.68 |
| Partner status: |  |  |
| - Partner, cohabiting vs none | 1.45 | 0.54-3.90 |
| - Partner, not cohabiting vs none | 2.84 | 0.65-12.40 |
| Working hours per hour | 1.01 | 0.98-1.03 |
| Functional limitations^a^ | 1.36 | 0.56-3.31 |
| Self-rated health^b^ | 1.80 | 0.93-3.49^†^ |
| Depressive symptoms^c^ | 0.32 | 0.04-2.51 |
| Cognitive impairment^d^ | 1.14 | 0.49-2.62 |

Abbreviations: OR=Odds Ratio; CI=Confidence Interval

^a^ Score >= 2

^b^ Less than good

^c^ Score Center for Epidemiologic Studies Depression scale >= 16

^d^ Score 9-item MiniMental State Examination <= 14

^†^ Significant at p<0.10; * Significant at p<0.05

**Supplement Table 2.** Correlations among the four health indicators (as continuous variables) and age: all 3 periods (n=413)

|  | Functional limitations | Self-rated health | Depressive symptoms | Cognitive ability |
| --- | --- | --- | --- | --- |
| Age | -0.07 | -0.05 | -0.11* | -0.04 |
| Functional limitations | 1.0 | 0.51** | 0.39** | -0.14* |
| Self-rated health |  | 1.0 | 0.46** | -0.07 |
| Depressive symptoms |  |  | 1.0 | -0.10* |
| Cognitive ability |  |  |  | 1.0 |

Notes: ** p<0.001; * p<0.05

**Supplement Table 3**. Retired workers: Association of baseline health (dichotomous) with reason for (partly) retirement at follow-up, 2019 and 2022 pooled (n=22); percentages are presented for participants stating each specific reason

|  | Functional limitations (%) | Less-than-good self-rated health (%) | Relevant depressive symptoms (%) | Cognitive impairment (%) |
| --- | --- | --- | --- | --- |
| Demands/health | 22^†^ | 56 | 0 | 0 |
| Lack of motivation | 11 | 67* | 0 | 0 |
| Private life | 0* | 31 | 6 | 6 |

Notes: * p<0.05; ^†^ p<0.10; the indication of significance is based on the comparison of participants stating a specific reason and participants not stating this reason.

**Supplement Table 4**. Continuing workers: Association of health (dichotomous) with reasons for wishing to exit earlier than SPA or to continue working, 2019 and 2022 pooled

|  | Functional limitations (%) | Less-than-good self-rated health (%) | Relevant depressive symptoms (%) | Cognitive impairment (%) |
| --- | --- | --- | --- | --- |
| *Reasons for wishing to exit earlier than SPA (n=63)* | | | | |
| Demands/health | 41* | 44* | 22* | 11 |
| Private life | 18 | 21 | 6 | 9 |
| Lack of motivation | 12 | 0* | 0 | 0 |
| Finances | 0 | 33 | 0 | 0 |
| **Total** | **25*** | **30*** | **11*** | **10** |
| *Reasons for wishing to continue working up to SPA (n=66)* | | | | |
| Motivation to work | 8 | 11 | 0 | 15 |
| Loyalty to others | 0 | 0 | 0 | 0 |
| Finances | 11 | 33 | 0 | 11 |
| **Total** | **11** | **14** | **0** | **15** |

Notes: * p<0.05; the indication of significance is based on the health comparison of participants stating a specific reason and participants not stating this reason; the bold entries present the health comparison of participants wishing to exit earlier and those who do not wish so.

**Supplement Table 5**. Continuing workers: Association of reducing working hours with reasons for wishing to exit earlier than SPA or to continue working

|  | Reduced working hours by >=4 hours (n=65) | Reduced working hours by <4 hours or did not reduce working hours (n=120) | |
| --- | --- | --- | --- |
| *Reasons for wishing to exit earlier than SPA* | | | |
| Demands/health | 15% | 18% |  |
| Private life | 29% | 20% |  |
| Lack of motivation | 12% | 3%* |  |
| Finances | 0 | 3% |  |
| *Reasons for wishing to continue working up to SPA* | | | |
| Motivation to work | 35% | 41% |  |
| Loyalty to others | 2% | 6% |  |
| Finances | 4% | 8% |  |

Notes: * p<0.05

**Supplement Table 6**. Associations (Odds Ratios, Confidence Intervals) of four dichotomous health indicators with continuing versus stopping paid work within 3 years in workers at baseline aged 61 to SRA minus 0.5 years. Logistic regression adjusted for age , sex, education, partner status, and period; each row is one model. In case of a significant interaction effect of health indicator*period, the ORs are presented for each period.

|  | OR | 95% CI |
| --- | --- | --- |
| Functional limitations^a^ | 0.54 | 0.20-1.44 |
| Self-rated health^b,†^: |  |  |
| - Period 1 | 0.93 | 0.22-3.88 |
| - Period 2 | 1.69 | 0.33-8.74 |
| - Period 3 | 0.13 | 0.04-0.40* |
| Depressive symptoms^c^ | 1.32 | 0.33-5.34 |
| Cognitive impairment^d^ | 1.39 | 0.49-3.95 |

^a^ Score >= 2

^b^ Less than good

^c^ Score Center for Epidemiologic Studies Depression scale >= 16

^d^ Score 9-item MiniMental State Examination <= 14

* p < 0.05

^†^ Interaction self-rated health * period significant at p=0.02

**Supplement Table 7**. Associations of baseline self-rated health among workforce participants with follow-up workforce participation in three time intervals during the COVID19 pandemic^a^

|  | N exit/total working at baseline | Continued working | Exited from workforce | p-value |
| --- | --- | --- | --- | --- |
| Self-rated health at beginning of interval (M,sd): |  |  |  |  |
| . 2018/19 – 1 March 2020 | 9^b^/76 | 2.0 (0.7) | 2.9 (0.7) | 0.005 |
| . 1 March – 1 October 2020 | 3/57 | 1.9 (0.6) | 2.3 (1.5) | 0.252 |
| . 1 October 2020 – 2021/22 | 10/61 | 2.1 (0.7) | 2.2 (0.4) | 0.528 |

^a^ The first interval starts with the general LASA-wave 2018/19; the last interval ends with the general LASA-wave 2021/22. In June 2020 and April 2021, two special COVID19-questionnaire were sent out, including questions about doing paid work. Response rates were 76% and 69%, respectively (Holwerda et al 2023). In each questionnaire, it was asked if the respondent did paid work just before the first and second pandemic waves, i.e., before 1 March 2020 and before 1 October 2020, respectively. Next, it was asked if the job changed, but the number of respondents exiting was too small (n=3 and n=1, respectively) to do further analyses.

^b^.Excluding 3 respondents who re-entered the workforce prior to 2021/22.

Holwerda TJ, Jaarsma E,· van Zutphen EM, Beekman ATF, Pan K-Y, van Vliet M, Stringa N, van den Besselaar JH, MacNeil‑Vroomen JL, Hoogendijk EO, Kok AAL. The impact of COVID‑19 related adversity on the course of mental health during the pandemic and the role of protective factors: a longitudinal study among older adults in The Netherlands. Social Psychiatry and Psychiatric Epidemiology 2023. https://doi.org/10.1007/s00127-023-02457-5

Note: the successive periods were of unequal length, so that the proportions exit cannot be compared.

Supplement Figure 1. Involvement in paid work: annual proportions in 3-year age groups, 2013-2023. Source: Statistics Netherlands (2024).

Supplement Figure 2. Proportion of hours worked per week in the years 2013-2023, workers in two age groups. Source: Statistics Netherlands (2024).
